# Leiomyoma of maxillary sinus

**DOI:** 10.4322/acr.2024.495

**Published:** 2024-06-13

**Authors:** Ravi Hari Phulware, Shalini Jain, Akash Dhiman, Arvind Ahuja, Arvind Kumar

**Affiliations:** 1 All India Institute of Medical Sciences, Department of Pathology & Laboratory Medicine, Rishikesh, Uttarakhand, India.; 2 Post Graduate Institute of Medical Education and Research & Dr RML Hospital, Specialist Doctor, Department Ear Nose Throat, New Delhi, India; 3 Post Graduate Institute of Medical Education and Research & Dr RML Hospital, Professor, Department of Pathology, New Delhi, India

Leiomyoma is a benign smooth muscle tumor that most commonly occurs in the uterus; however, it can also manifest in various extragenital locations, including the maxillary sinus.^1,2^ While leiomyomas of the maxillary sinus are occasional, they present unique diagnostic and management challenges. The maxillary sinus, one of the paranasal sinuses in the facial bones, is typically associated with sinusitis or odontogenic infections.^1^ Therefore, clinicians primarily consider inflammatory or neoplastic etiologies when encountering a mass within the maxillary sinus. Among the possible neoplastic causes, leiomyoma is a less frequently encountered entity, but it should be considered in the differential diagnosis.^1,2^

Understanding the clinical presentation, radiological features, and appropriate management strategies for leiomyoma of the maxillary sinus is crucial for accurate diagnosis and effective treatment. This case report aims to contribute to the existing medical knowledge by presenting a unique case of leiomyoma arising in the maxillary sinus, discussing the diagnostic approach treatment options, and highlighting any noteworthy findings.^2,3^

Leiomyoma of the maxillary sinus can present with various symptoms, including nasal obstruction, facial pain or pressure, recurrent sinusitis, epistaxis (nosebleeds), or a palpable mass in the maxillary region.^1,3^ However, these symptoms are non-specific and can be mistaken for other sinus entities. Therefore, it is essential to consider leiomyoma as a differential diagnosis in patients with such clinical presentations. Diagnosing leiomyoma of the maxillary sinus can be challenging due to its rarity and overlapping symptoms with other sinus entities. Imaging studies, such as compute tomography (CT) or magnetic resonance imaging (MRI), play a crucial role in identifying the presence, location, and extent of the tumor.^3,4^ However, a definitive diagnosis requires a histopathological examination of the excised tissue. When encountering a maxillary sinus mass, the differential diagnosis includes various benign and malignant conditions. Potential differentials include inverted papilloma, angiofibroma, Schneiderian papilloma, mucocele, and even malignancies like sinonasal carcinoma or sarcoma. Proper evaluation, including clinical, radiological, and histopathological assessment, is necessary to differentiate leiomyoma from other lesions.^4,5^

Khanna et al.^1^ reported the case of a leiomyoma originating in the maxillary sinus in a 55-year-old female. The patient presented with facial pain and nasal obstruction. The CT revealed a well-defined mass in the maxillary sinus. Surgical excision was performed, and the histopathological examination confirmed the diagnosis of leiomyoma. Rana et al.^2^ presented a case of a 37-year-old male with a leiomyoma in the maxillary sinus. The patient complained of nasal obstruction and recurrent sinusitis. The CT imaging showed a polypoidal mass in the maxillary sinus, and endoscopic sinus surgery was performed to remove the tumor. Histopathological examination confirmed the diagnosis of leiomyoma. In a retrospective study by Li et al.^3^ seven cases of leiomyoma arising from the maxillary sinus were analyzed. The study highlighted these patients' clinical features, radiological findings, and treatment outcomes. The authors emphasized the importance of considering leiomyoma as a potential diagnosis when evaluating maxillary sinus masses. Khan et al.^4^ reported a case of leiomyoma originating from the maxillary sinus in a 50-year-old male. The patient presented with nasal obstruction, facial pain, and recurrent epistaxis. Imaging studies revealed a soft tissue mass in the maxillary sinus, and endoscopic sinus surgery was performed for complete excision. Histopathological examination confirmed the diagnosis of leiomyoma. Another case report by Nandedkar et al.^5^ described a rare presentation of leiomyoma in the maxillary sinus in a 24-year-old male. The patient presented with nasal obstruction and was found to have a mass in the maxillary sinus on imaging. Endoscopic sinus surgery was performed, and the tumor was completely excised. Histopathological examination confirmed the diagnosis of leiomyoma.

These case reports and studies collectively highlight the variable clinical presentations, radiological features, and successful management strategies for the leiomyoma of the maxillary sinus. Due to the rarity of this condition, a high index of suspicion is required for accurate diagnosis, and surgical excision is the mainstay of treatment. Further research and case studies are needed to expand our knowledge and improve patient outcomes in this rare entity.^3,5^

The mainstay of treatment for leiomyoma of the maxillary sinus is surgical excision. The approach can vary depending on the size and location of the tumor. Endoscopic sinus surgery (ESS) is often employed as a minimally invasive technique for complete resection.^2,3^ In certain cases, an open surgical approach may be required for more extensive tumors. Surgery aims to achieve complete excision while preserving the surrounding structures and achieving symptomatic relief. Leiomyoma of the maxillary sinus has a favorable prognosis due to its benign nature. Complete surgical excision usually leads to the resolution of symptoms and low recurrence rates. Long-term follow-up is important to monitor for any recurrence or complications.^1,2^ It is important to note that the literature on leiomyoma of the maxillary sinus is limited to case reports and small studies due to its rarity. Further research and larger studies are needed to gather more data on this condition's clinical characteristics, optimal treatment approaches, and long-term outcomes. Postoperative care typically involves nasal irrigation, pain management, and antibiotics. Regular follow-up visits are essential to monitor the patient's recovery, assess for any signs of recurrence, and manage any postoperative complications.^2,4^ The prognosis of leiomyoma of the maxillary sinus is generally favorable. Since leiomyomas are benign tumors, they do not have the potential to metastasize or invade adjacent structures. With complete surgical excision, most patients experience symptom resolution and have low recurrence rates.^4,5^

In conclusion, leiomyoma of the maxillary sinus is a rare entity that requires consideration in the differential diagnosis of maxillary sinus masses. Despite the diagnostic challenges, accurate evaluation through clinical assessment, imaging studies, and histopathological examination is crucial for appropriate management. Surgical excision remains the primary treatment modality, with a favorable prognosis and low recurrence rates.

**Figure 1:**
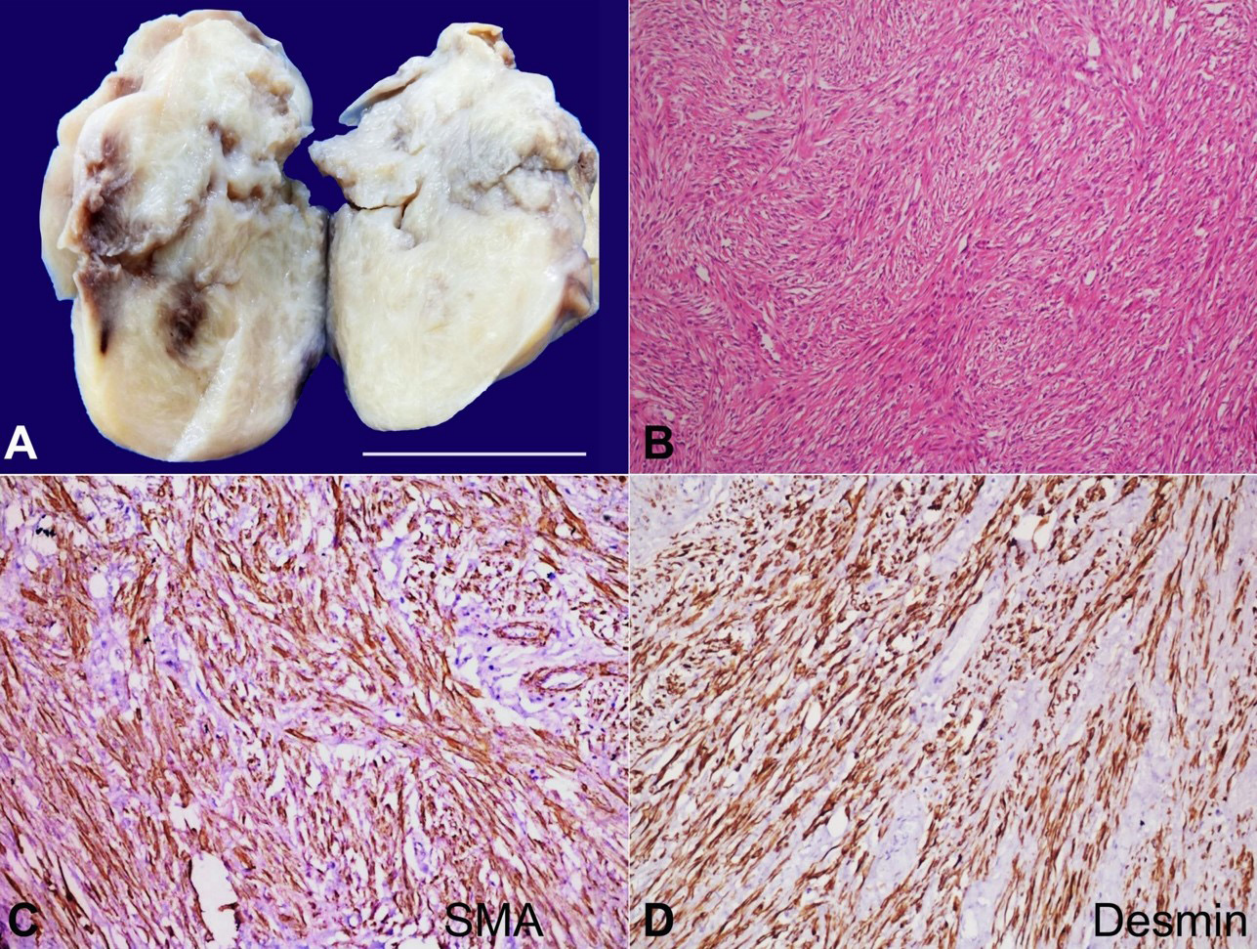
**A –** Gross photomicrograph shows a greyish white to tan-white well circumscribed but nonencapsulated tumor. The cut surface shows firm, whorled surface areas with hemorrhage (scale bar= 5 cm); **B –** shows a tumor composed of interlacing fascicles of smooth muscle bundles (H&E, 40X); **C –** Immunohistochemical reaction for smooth muscle actin (SMA) demonstrating smooth muscle bundles and vessel walls (100X); **D –** Immunohistochemical reaction for Desmin shows diffuse cytoplasmic positivity (100X).

Figure 1 refers to a 45-year-old female patient who presented to the otolaryngology clinic complaining of progressive nasal obstruction and intermittent dull facial pain on the right side. She reported that her symptoms had been gradually worsening over the past six months. There were no associated symptoms such as epistaxis, nasal discharge, or changes in smell. The patient had no significant medical history and did not report any history of previous sinus infections or allergies. She had no history of smoking or exposure to environmental toxins. On physical examination, tenderness was on palpation over the right maxillary sinus area. The nasal passages appeared patent, with no visible polyps or signs of inflammation. The rest of the head and neck examination, including the oral cavity, was unremarkable. A flexible nasal endoscopy revealed a bulging mass in the right middle meatus obstructing the ostium of the maxillary sinus. The rest of the nasal cavity and other sinus ostia appeared normal. A computed tomography (CT) scan of the paranasal sinuses was obtained, which demonstrated a well-defined, non-enhancing soft tissue mass filling the right maxillary sinus. There were no signs of erosion into surrounding structures or evidence of distant metastasis.

Based on the clinical presentation and imaging findings, a provisional diagnosis of leiomyoma of the maxillary sinus was made. The patient was counseled about the benign nature of the tumor and the recommended treatment options. The patient underwent endoscopic sinus surgery (ESS) for complete excision of the leiomyoma. During the procedure, the tumor was identified and carefully dissected from the surrounding sinus mucosa. Hemostasis was achieved, and the surgical site was thoroughly irrigated.

Histopathological examination of the excised specimen confirmed the diagnosis of leiomyoma, showing spindle-shaped cells with cigar-shaped nuclei arranged in fascicles. No malignant features or atypical mitotic figures were observed. The patient had an uneventful postoperative recovery with the resolution of her symptoms. She was followed up regularly to monitor for any signs of recurrence or complications. During the follow-up visits, the patient remained asymptomatic, with no signs of tumor recurrence or associated complications.
